# Editorial

**Published:** 1842-03-19

**Authors:** 


### THE MEDICAL EXAMINER.

### PHILADELPHIA, MARCH 19, 1842.

### MISCELLANEOUS NOTICES.

*Medical Schools.*—During the session just concluded, there have been at the University of Pennsylvania, 362 students ; at the Jefferson College of Philadelphia, 209 students and 61 graduates ; at the Pennsylvania Medical College, 83 students and 29 graduates ; at the Albany Medical College, 101 students and 27 graduates ; at the Geneva Medical College, 211 students; at the Medical Department of Yale College, 47 students and 19 graduates.

*Baltimore College of Dental Surgery.*—At the second annual commencement of this useful institution, the degree of Doctor of Dental Surgery was conferred upon three graduates, besides several honorary degrees. An appropriate valedictory address was delivered by Professor W. R. Handy.

*Physiological Temperance Society of the Medical Institute of Louisville.*—The object of this society, besides the suppression of intemperance, is the investigation of its causes, consequences, and remedies, on physiological and pathological principles, including other stimulants and narcotics than alcohol. Professor Daniel Drake is the President of the new society—an earnest that the proposed investigations will be conducted with spirit and ability. Among the corresponding members we notice the name of Dr. John Bell, of Philadelphia, a well-known advocate of the cause.

*New Quarterly Medical Journal in Boston.*—A new journal is announced, under the title of the "New England Quarterly Journal of Medicine and

Surgery," to be edited by Drs. Ware and Parkman, of Boston. Fromt he character of the editors, and the promises of aid under which they take the field, the newjournal is likely to be a very valuable addition to the corps. We cordially wish it success.

*The Cortlandville affair, once more.*—The question of amputation in this case, the Cortland county doctors seem determined to keep alive. A correspondent of the Boston Med. and Surg. Journ. under date of February 21st, writes that the patient " is again in the alms-house and very feeble. The limb," he says, "has never been healed"—and he intimates that "an amputation may yet be necessary" ! Upon loose anonymous statements like this, no reliance is to be placed, but the *intimation* is not without meaning.

